# Survival of Group A Streptococcus (GAS) is Enhanced Under Desiccated Culture Conditions

**DOI:** 10.1007/s00284-020-01967-8

**Published:** 2020-04-02

**Authors:** Leonhard Menschner, Uta Falke, Peter Konrad, Nicole Toepfner, Reinhard Berner

**Affiliations:** Department of Pediatrics, University Hospital Carl Gustav Carus, Technische Universität Dresden, Fetscherstrasse 74, 01307 Dresden, Germany

## Abstract

*Streptococcus pyogenes* or Group A Streptococcus (GAS) infections are the leading cause of bacterial tonsillopharyngitis. The bacterium can survive and persist within the human host for a long time as it is observed in up to 40% of the population who are considered as carriers. Recurrent tonsillopharyngitis is a particular problem in children which is caused either by relapses due to failed bacterial clearance or by reinfection. A prolonged survival in tonsillar crypts or on inanimate surfaces might be sources for reinfection. We therefore examined 64 clinical GAS isolates from children with tonsillopharyngitis for their long-term survival under either liquid or desiccated culture conditions. After 6 weeks, the overall GAS survival rate was 400-fold increased under desiccated culture conditions compared to liquid culture conditions, but varied depending on the *emm*-type between 20-fold (*emm*4) and 14000-fold (*emm*3). The survival rates of isolates from *emm75* were significantly lower which is probably due to their production of hydrogen peroxide up to fatal doses. No hydrogen peroxide production could be detected for other *emm*-types. Furthermore, 11 isolates from patients with recurrent tonsillopharyngitis were compared to isolates of the same *emm*-type from patients with single episodes of tonsillopharyngitis. A significant elevated pH value and an increased survival rate for isolates from patients with recurrent infections were observed. In conclusion, significant differences in long-term survival of different GAS isolates as well as survival under desiccated culture conditions might contribute to both failed bacterial clearance and reinfection in patients with recurrent tonsillopharyngitis.

**Electronic supplementary material:**

The online version of this article (10.1007/s00284-020-01967-8) contains supplementary material, which is available to authorized users.

## Introduction

*Streptococcus pyogenes*, or Group A Streptococcus (GAS), is an important human pathogen which causes a broad range of infections such as pharyngitis, impetigo, cellulitis, scarlet fever, pneumonia, sepsis and streptococcal toxic shock syndrome [1]. The global burden of GAS-related disease is estimated at over 616 million incident cases of pharyngitis per year and a prevalence of at least 18.1 million cases of invasive diseases [2]. GAS is a predominant human pathogen which inhabits the oropharynx representing its primary reservoir. The GAS carriage rate is highest among children with estimated 2 to 17% [3–5] but shows seasonal and socio-epidemiological variations. One explanation for the maintenance of carrier state is the capability of GAS to form biofilms in the ground of tonsillar crypts [6, 7]. This nutrient-limited niche can also serve as reservoir for recurrent tonsillitis but requires GAS to persist over a prolonged period of time [8]. Although transmission occurs usually by airborne droplets through human-to-human interaction, reacquisition from environmental surfaces cannot be excluded as viable GAS could be isolated, e.g., from toys or toothbrushes [9, 10]. A prolonged persistence of GAS was proven for up to 6 month on dry inanimate surfaces or for over 1 year under liquid culture conditions [11–13]. Starvation and desiccation have to be regarded as different processes since the loss of water results in a full metabolic arrest whereas starvation does not [14, 15].

Long-term survival is important for GAS under several circumstances but only a limited number of isolates and settings were studied so far. Considering the broad range of clinical presentations from asymptomatic GAS carriers to patients with life-threatening diseases, the GAS survival rate might even vary between different isolates. Here, we characterize the long-term survival of 64 GAS tonsillopharyngitis isolates of 8 different *emm*-types under liquid and desiccated culture conditions with special attention to isolates from patients with recurrent GAS infections.

## Materials and Methods

### Bacterial Isolates and Cultivation

All 64 clinical GAS isolates were obtained from children between 1 and 13 years of age at the University Hospital Freiburg, Germany between 2006 and 2012. All patients were diagnosed with GAS tonsillopharyngitis and 11 out of them suffered from recurrent episodes of GAS tonsillopharyngitis. All GAS isolates were grown on Columbia Agar plates supplemented with 5% sheep blood (bioMérieux, Nürtingen, Germany) at 37 °C and 5% CO_2_.

### *emm*-typing

The M protein gene (*emm*) encodes for a cell surface protein which is responsible for more than100 GAS M serotypes. The *emm*-typing of the 5’ variable region was carried out according to the protocols by the CDC (https://www.cdc.gov/streplab/protocol-emm-type.html). The PCR products were outsourced for sequence analysis (Seqlab, Goettingen, Germany). The obtained sequences were compared with the sequences in the *emm*-type database available on the CDC website (https://www2a.cdc.gov/ncidod/biotech/strepblast.asp).

### Bacterial Survival Kinetics

Overnight cultures in Brain Heart Infusion (BHI; BD, Heidelberg, Germany) were inoculated from single colonies on agar plates streaked from glycerol stocks. For survival kinetics, pre-warmed BHI medium was inoculated with cells from an exponentially growing overnight culture and further cultivated at 37 °C and 5% CO_2_ until the stationary phase was reached. At this point, the first sample was examined and aliquots were prepared as liquid culture in falcon tubes or as desiccated cultures in 24-well plates. Desiccation was achieved by air drying under sterile conditions. Survival was monitored by colony plating following previous rehydration for desiccated samples. The incubation period ranged from 1 to 42 days and was performed at room temperature protected from light. 8 GAS isolates per *emm*-type were analyzed (*emm1*, *emm2*, *emm3*, *emm4*, *emm12*, *emm28*, *emm75*, *emm89*).

### Analysis of pH and Hydrogen Peroxide Production

Aliquots of liquid cultures were centrifuged at 8.500 rpm for 5 min and the pH of the supernatant was determined (pHenomenal 1000L; VWR, Darmstadt, Germany). To investigate whether the pH had an impact on the long-term survival, the survival kinetic experiment was repeated for six isolates (three *emm1* and three *emm75*) in media with 10 mM HEPES buffer. In HEPES buffered media, the supernatant of these six isolates had a pH of ~ 5.7 compared to a pH of < 5.5 in unbuffered BHI media. The relative amount of hydrogen peroxide (H_2_O_2_) produced by each GAS culture was determined semi-quantitatively using the previously described Prussian-blue agar (PB agar) [16]. In brief, 100 µl of supernatant from each culture were spotted on PB agar and incubated for 10 min. The staining was compared to standard concentrations of hydrogen peroxide.

## Results

### Survival Rate of GAS Declines Over Time

**Fig. 1:**
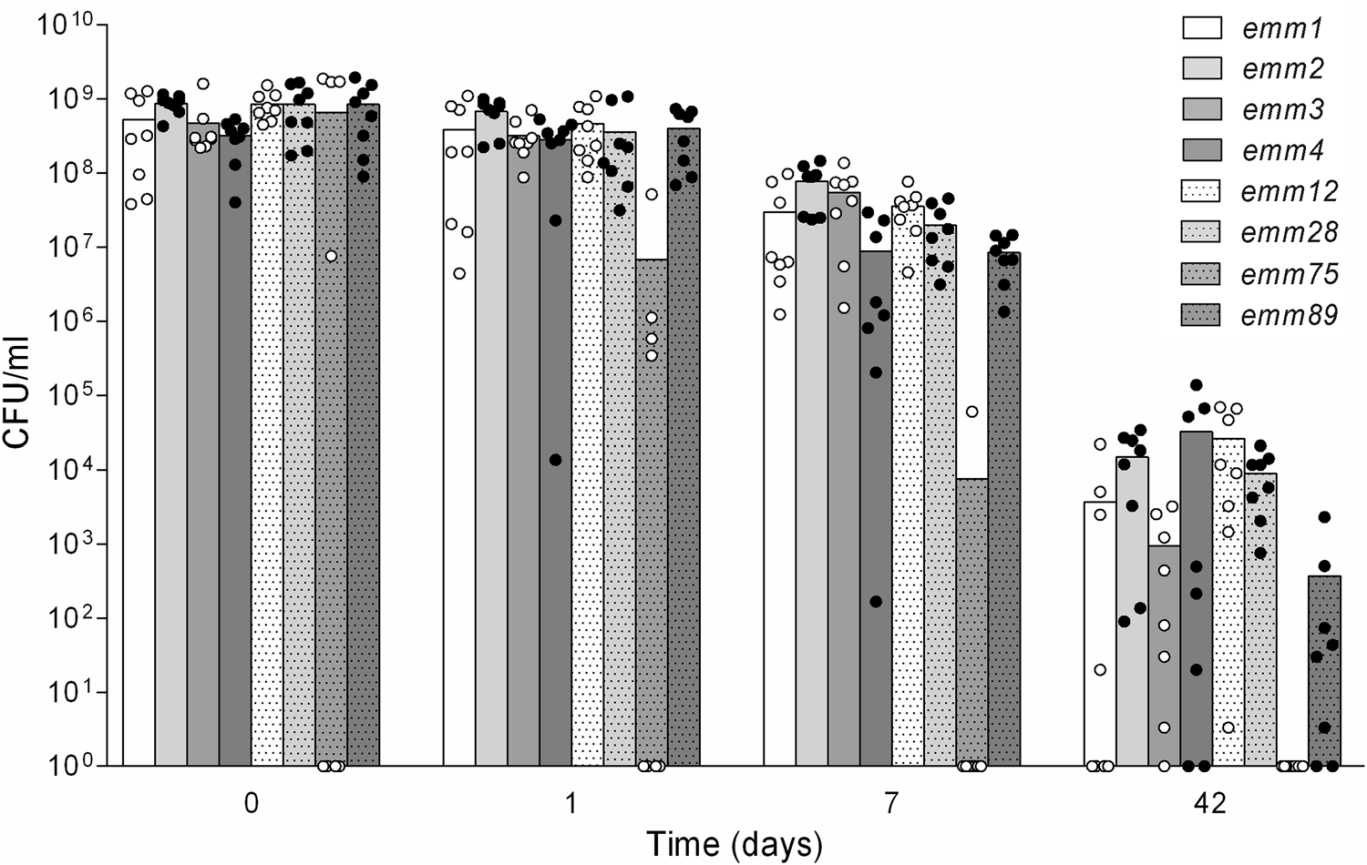
Stationary-phase survival kinetics of different clinical GAS isolates in BHI. Overall, 64 clinical GAS isolates of 8 different *emm*-types were grown in BHI until stationary phase (day 0). Further cultivation was performed for another 6 weeks: CFU were determined on Columbia blood agar plates at time points day 0, day 1, day 7 and day 42. Dots in the graph represent the mean of three experiments per isolate, whereas the bars represent the mean per *emm*-type: a significant effect over time (p < 0.0001) was proven by two-way analysis of variance. A significantly decline was observed for *emm75*-isolates (Multiple comparisons by Kruskal–Wallis test)

The survival of all 64 clinical isolates of 8 different *emm*-types in the complex medium BHI was observed over an extended cultivation period of 6 weeks. All isolates reached the stationary phase but no viable cells could be detected for 4 out of 8 *emm75*-isolates. The remaining 60 isolates had initially about 10^8^ to 10^9^ colony forming units per ml (CFU/ml) which declined exponentially over time (Fig. 1). The variation within each isolate (triplicate) was low but differs within each *emm*-type (8 isolates). Statistical analysis with two-way analysis of variance revealed significant effects over time (*P*<0.0001) and a significantly increased decline was observed for *emm75*-isolates (Multiple comparisons by Kruskal–Wallis test).

### Culture pH Stays Constant Over Time

The pH for liquid cultivated bacteria was determined at all time points. The pH only varied slightly within a given isolate, and the majority had a pH above the critical value of 5.5 [17]. 11 isolates had a pH<5.5 including three *emm1*-isolates (pH=5.43) and three of the four *emm75*-isolates (pH=5.44) that did not survive the initial stationary phase. In the survival kinetic experiment with buffered media, an improved growth with a higher optical density could be detected for the three *emm75*-isolates but it did not prevent their decay in the initial stationary phase. For the three *emm1*-isolates, the higher pH only slightly increased their cell numbers (Online Resource 1).

### Decay by Fatal Production of H_2_O_2_

PB-formation analysis was used for semi-quantitative detection of H_2_O_2_ in culture supernatants. For 8 isolates a H_2_O_2_ production was detected and they all belonged to the *emm*-type 75.

### Desiccation Improves Long-Term Survival *emm*-type Dependent

**Fig. 2:**
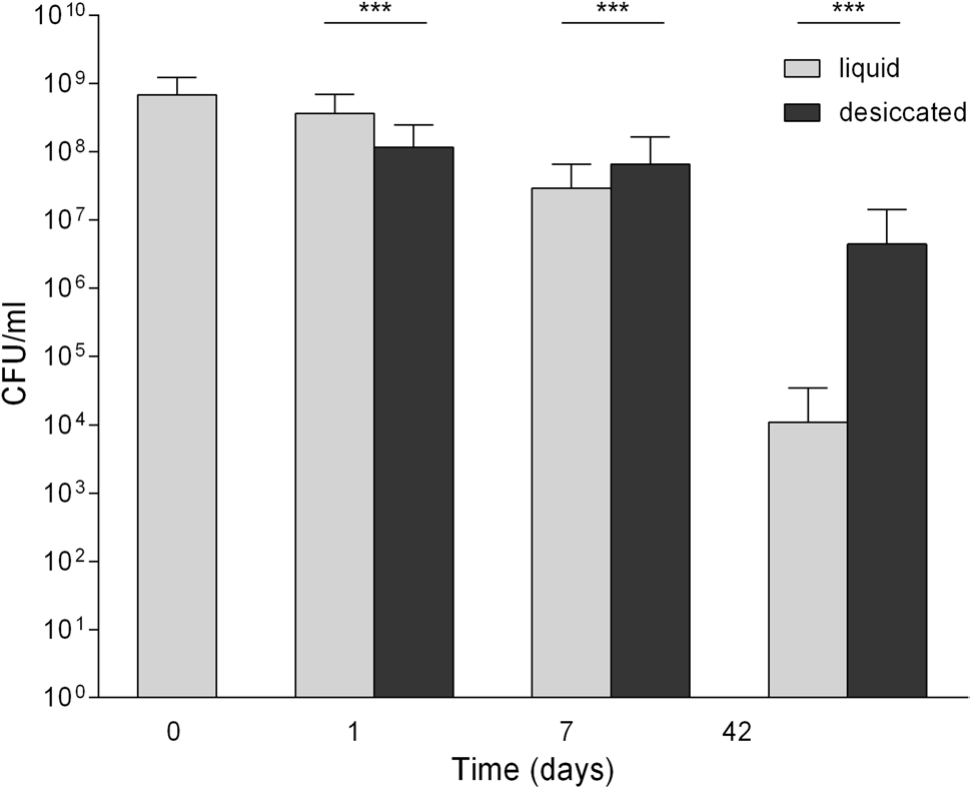
Effect of desiccation on the stationary-phase survival kinetics of GAS isolates. Overall, 64 clinical GAS isolates were grown in BHI. Culture aliquots of each isolate were transferred to wells and desiccated (black bar) or further cultivated in BHI (gray bar). The mean and standard deviation of CFU is depicted from three experiments per isolate. Statistical analysis was performed by Wilcoxon signed rank test: ***P < 0.001

A threefold decrease in survival was observed after 1 day of desiccation compared to liquid cultivation (Fig. 2). The situation changed at day 7 where survival improved in the desiccated culture; at day 42 even a 400-fold overall difference was observed. However, this effect varied among the tested *emm*-types: *emm4* 20x, *emm12* 108x, *emm2* 157x, *emm89* 710x, *emm28* 786x, *emm1* 2218× and *emm3* 14779x, whereas no CFU could be detected for *emm75* under liquid cultivation after 42 days. A significant difference between culture conditions (*P*<0.0012 for 7d and *P*<0.0002 for 42d) and *emm*-type (*P*<0.0001 for 7d and *P*<0.0341 for 42d) was proven by a two-way analysis of variance.

### Isolates of Recurrent Tonsillopharyngitis Show Increased pH and Survival

The survival rate was increased in 11 GAS isolates from patients with recurrent tonsillopharyngitis compared to GAS isolated from patients with single episodes of tonsillopharyngitis (Fig. 4). With respect to the *emm*-type variation described above, both groups consisted of the same *emm*-types, in particular *emm1*, *emm2*, *emm12*, *emm28* and *emm89*. The supernatant of GAS isolates from patients with recurrent tonsillopharyngitis also exhibited a significant higher pH at all time points being 5.9 compared to 5.6 (Fig. 4). Statistical analysis was performed by Mann–Whitney test.

## Discussion

This study describes the long-term survival of a representative number of GAS isolates. Shelburne et al. showed a prolonged survival of GAS in human saliva for 28 days at a rate similar to complex media used in this study [18]. Upon reaching the stationary phase, the tested isolates had an initial CFU of about 10^8^ to 10^9^ cells/ml, which is similar to previously published results [12, 17, 19]. Overall, a decline in survival over time was observed but interestingly at 42 days of cultivation a significantly improved survival rate was detected under desiccated conditions compared to liquid cultivation (Fig. 2).

Under liquid culture conditions GAS stay metabolically active and consume lactate and amino acids after glucose exhaustion to maintain their basal metabolism [20]. An increased use of the pyruvate pathway is described to be beneficial for a prolonged GAS survival [20]. The metabolic pathways used by GAS strongly depends on the availability of oxygen which is limited deep in tonsillar crypts or in biofilms [21] but not in the pharyngeal mucosa. Under anaerobe conditions, the pyruvate formate lyase together with the mixed acid fermentation plays an important role as they provide ATP without NADH production [20]. However, GAS possesses a very effective NADH oxidase which regenerates NAD under aerobe conditions [22, 23]. This is especially important as exceeded levels of NADH can reduce Fe^3+^ to Fe^2+^ which leads to the risk of the Fenton reaction [22].

**Fig. 3:**
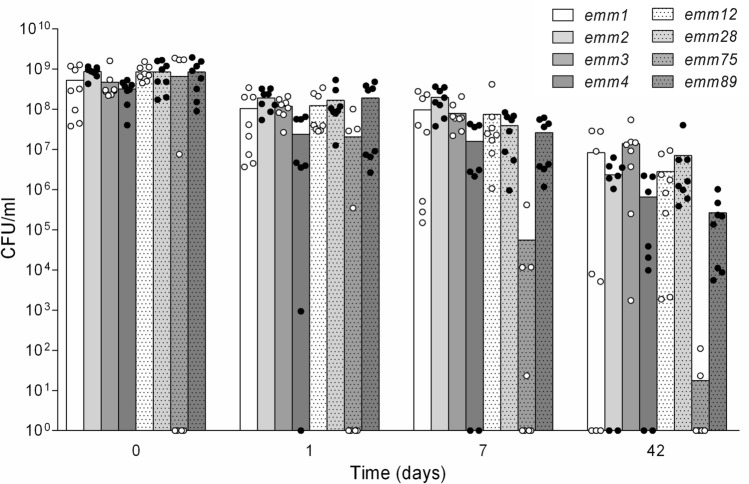
Stationary-phase survival kinetics of different clinical GAS isolates after desiccation. Overall, 64 clinical GAS isolates of 8 different *emm*-types were grown in BHI until stationary phase (day 0). The cultures were air dried under sterile conditions and the CFU were determined on Columbia blood agar plates over a period of 6 weeks at time points day 0, day 1, day 7 and day 42. Dots in the graph represent the mean of three experiments per isolate, whereas the bars represent the mean per *emm*-type: a significant effect over time (*P* < 0.0001) was proven by two-way analysis of variance. A significantly decline was observed for *emm75*-isolates (Multiple comparisons by Kruskal–Wallis test)

In this study, we showed that most GAS isolates are able to survive under desiccated conditions with a high cell count (Fig. 3). This proves the ability of GAS to persist on dry surfaces for a prolonged time which creates opportunities for infection or reinfection, since successful isolation of GAS from, e.g., toys and toothbrushes has been described [9, 10]. Although viability and vitality have to be considered separately, the infectiousness was proven recently for desiccated strains of *S. pneumoniae* [15]. Therefore, environmental surfaces might serve as reservoir for GAS and play a role for infection as well as reinfection.

The availability of water has huge impacts on living cells. The loss of water leads to cell shrinking, increased viscosity, salt precipitation and finally to a full metabolic arrest [14]. The replacement of water with sucrose or other polyhydroxyl compounds is essential, as proteins would otherwise unfold by losing their hydrophilic/hydrophobic characteristics [24]. This is accomplished by osmotic or cryoprotective substances which are induced upon desiccation by either increased salt concentration or cooling through water evaporation [25]. With an arrested metabolism no starvation induced cell death occurs and the chemical stability of DNA is a major factor for cell survival. DNA repair upon rehydration is therefore a critical step that has to overcome strand breaks by ultraviolet radiation or DNA modifications by radicals. These radicals are produced by metal-catalyzed or Maillard reactions [14].

For the *emm*-types 2 and 4, a chromosomal island is described which integrates into the genome upon the stationary phase [26, 27]. This integration disrupts the transcription of genes for DNA mismatch and base excision repair [26, 27] and might be the reason for the decay of some isolates of the *emm*-types 1, 2, 4 and 89 after 42 days under desiccation. Furthermore, we found that all isolates with the *emm*-type 75 produced sufficient amounts of H_2_O_2_. Four isolates already deceased upon entry into the stationary phase (day 0), whereas the remaining 4 isolates declined rapidly and no viable cells could be detected after 42d of liquid cultivation (Fig. 1). This might be the result of the production of fatal amounts of H_2_O_2_ [28, 29] rather than a pH-shift below the critical value of 5.5 [12, 17] as we could show that *emm75*-isolates also deceased in bufferd media with a pH of 5.7. Until now the role of H_2_O_2_ is unclear, as it is unknown if lethal levels are reached in vivo or if these levels are decomposed by the host or catalase-positive biofilm partners [30]. Hypotheses range from metabolic advantage by aerobic utilization of lactate [31] to increased invasiveness through H_2_O_2_ [32].

Overall a discrepancy between different GAS isolates and *emm*-types could be detected which is supposed to contribute to their different survival strategies (Fig. 1, 3). This is of special interest as all examined isolates originated from patients with tonsillopharyngitis and they are among the 10 most common *emm*-types that contribute to pharyngeal disease in established market economy countries [33]. This indicates a specific adaption strategy to the environmental niche of the human oropharynx. Here, we detected a growth decay for some isolates of the *emm*-types 4 and 75 but they might be able to overcome the starvation in vivo by acquiring new resources as an increased invasion capacity into human nasopharyngeal cells (Detroit 562 cells) has been described for these particular *emm*-types [34].

On the other hand, the survival in nitrogen limited media might be decreased because GAS is unable to produce ammonia which is necessary to prevent a pH-shift below the critical value of 5.5 [19, 22]. Interestingly three *emm*-type 1 isolates from this study survived for 42 days with a constant pH of 5.43. This observation contradicts the generalization of a critical pH value [12, 17] although the mechanism behind remains unknown.

**Fig. 4:**
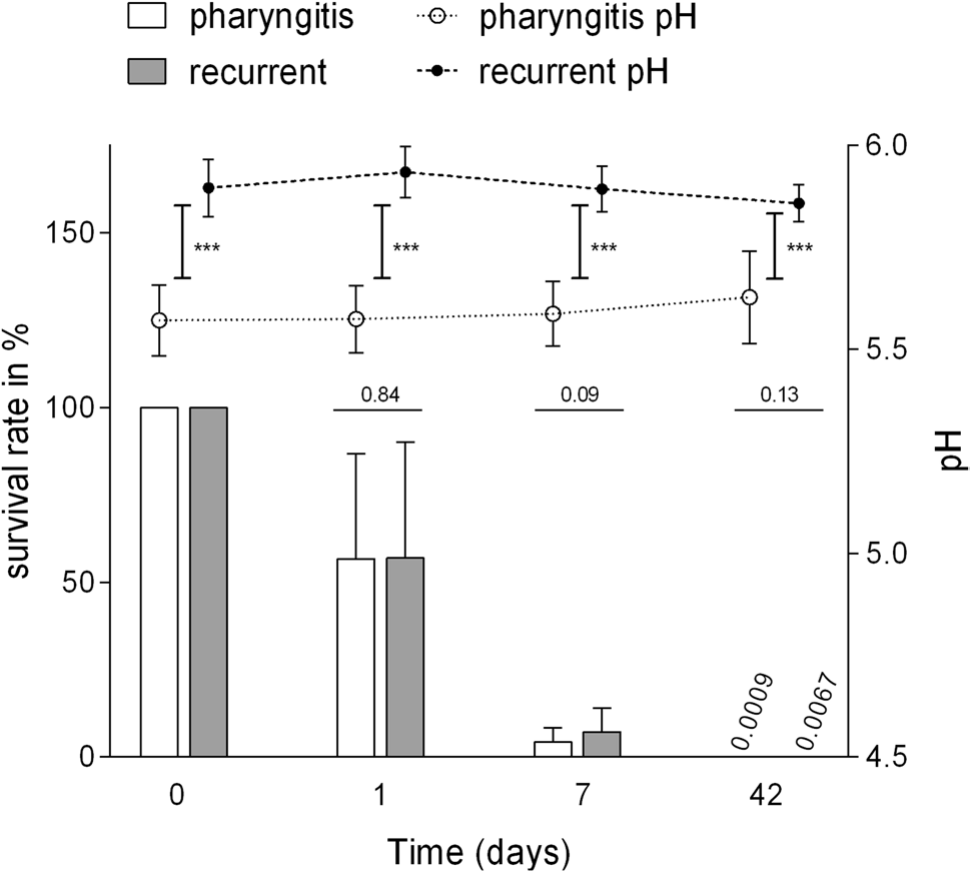
Increased pH values and long-term survival rates of GAS isolates from patients with recurrent tonsillopharyngitis. Long-term survival rates in BHI of 11 GAS isolates of patients with recurrent tonsillopharyngitis (gray bars) compared to GAS isolates of the same *emm*-type from patients with single episodes of tonsillopharyngitis (white bars). Values for CFU and culture pH represent the mean and standard deviation of three experiments per isolate at time points day 0, day 1, day 7 and day 42. The pH values in the culture of GAS isolated from patients with recurrent tonsillopharyngitis (black circle) and those from patients with single episodes of tonsillopharyngitis (white circle) differ significantly at all time points: ***P < 0.0001. Statistical analysis was performed by Mann—Whitney test

Furthermore, our study revealed an increased pH around 5.9 compared to 5.6 in cultures of GAS isolated from patients with recurrent tonsillopharyngitis compared to isolates of patients with single episodes of tonsillopharyngitis (Fig. 4). This elevated pH was accompanied by an increased survival rate but might have a stronger effect on the biofilm composition. The acquisition of β-lactamase producing biofilm partners [35] or a thick extracellular matrix which leads to poor penetration of antibiotics [36] are supposed to contribute to penicillin treatment failure which vice versa may lead to recurrent tonsillopharyngitis [37].

In conclusion, we could show isolate and *emm*-type-dependent differences of GAS long-term survival which point towards different GAS adaption strategies to the human oropharynx. We found an elevated pH as well as an increased survival for GAS which were isolated from patients with recurrent tonsillopharyngitis, indicating a particular adaption strategy. Furthermore, we observed an improved desiccation tolerance for GAS which indicates that environmental surfaces might contribute as a source for infection or reinfection.

## Electronic supplementary material

Below is the link to the electronic supplementary material.

Supplementary file1 (XLSX 41 kb)

## Abbreviations

GAS: Group a streptococcus
H_2_O_2_: Hydrogen peroxide
BHI: Brain Heart Infusion
CFU: Colony forming units
PB: Prussian Blue
