# Correction to: Survival of Group A Streptococcus (GAS) is Enhanced Under Desiccated Culture Conditions

**DOI:** 10.1007/s00284-021-02565-y

**Published:** 2021-06-28

**Authors:** Leonhard Menschner, Uta Falke, Peter Konrad, Nicole Toepfner, Reinhard Berner

**Affiliations:** Department of Pediatrics, University Hospital Carl Gustav Carus, Technische Universität Dresden, Fetscherstrasse 74, 01307 Dresden, Germany

## Correction to: Current Microbiology (2020) 77:1518–1524 10.1007/s00284-020-01967-8

The article “Survival of Group A Streptococcus (GAS) is Enhanced Under Desiccated Culture Conditions”, written by Leonhard Menschner, Uta Falke, Peter Konrad, Nicole Toepfner, Reinhard Berner, was originally published electronically on the publisher’s internet portal on 2 April 2020 without open access. With the author(s)' decision to opt for Open Choice the copyright of the article changed on 4 June 2021 to © The Author(s) 2020 and the article is forthwith distributed under a Creative Commons Attribution 4.0 International License, which permits use, sharing, adaptation, distribution and reproduction in any medium or format, as long as you give appropriate credit to the original author(s) and the source, provide a link to the Creative Commons licence, and indicate if changes were made. The images or other third party material in this article are included in the article’s Creative Commons licence, unless indicated otherwise in a credit line to the material. If material is not included in the article’s Creative Commons licence and your intended use is not permitted by statutory regulation or exceeds the permitted use, you will need to obtain permission directly from the copyright holder. To view a copy of this licence, visit http://creativecommons.org/licenses/by/4.0.

The original article has been corrected.

